# Transcaruncular Approach With Orbital Protection for Resection of Sinonasal Lesions: How I do it

**DOI:** 10.1177/19458924251364570

**Published:** 2025-08-01

**Authors:** Jakob L Fischer, Kelsey A Roelofs, Persiana S. Saffari, Jeff D Suh, Daniel B Rootman, Robert A Goldberg, Jivianne T Lee

**Affiliations:** 1 Department of Head and Neck Surgery, 8783 University of California, Los Angeles, CA, USA; 2 Department of Ophthalmology and Visual Sciences, 195981 University of Alberta, Edmonton, Canada; 3 Department of Ophthalmology, 22130 Bascom Palmer Eye Institute, University of Miami, Miami, FL USA; 4 Division of Orbital and Ophthalmic Plastic Surgery, 43352 Jules Stein Eye Institute, University of California, Los Angeles, CA, USA

**Keywords:** transcaruncular approach, orbital protection, sinonasal malignancy, sinonasal mass, endoscopic sinus surgery, orbital shield, lamina papyracea, anterior ethmoidal artery, skull base neoplasm, surgical techinques

## Abstract

**Background:**

Minimally invasive techniques for the resection of sinonasal masses have become increasingly important over the past few decades. Sinonasal disease involving the lamina papyracea remains difficult to manage given the risk of injury to critical orbital structures and hemorrhage from nearby vessels.

**Objective:**

Detail the transcaruncular approach with orbital protection for the resection of benign and malignant sinonasal pathologies.

**Methods:**

Description of surgical technique and presentation of 2 representative cases that were successfully managed with this surgical technique.

**Results:**

The transcaruncular approach involves incising the lateral 1/3 of the caruncle in a vertical plane between the upper and lower puncta. Dissection is then carried through the retrocaruncular fascia posterior to Horner's muscle to the posterior lacrimal crest along the medial orbital wall. Dissection can then be performed in a subperiosteal or supraperiosteal plane with subsequent ligation of the anterior ethmoidal artery. Once dissected, a nylon sheet used for orbital reconstruction and colored orbital shield can then be placed to aid in protection and visualization or orbital contents during endonasal tumor resection.

**Conclusion:**

The transcaruncular approach with orbital protection provides intraoperative protection of the orbital contents, allowing for safer removal of the mass irrespective of integrity of the lamina papyracea.

## Introduction

Improvements in endoscopic surgical instrumentation have fostered the development of minimally invasive techniques for sinonasal tumor resection. When pathology erodes through the lamina papyracea, caution must be exercised to avoid damage to critical orbital structures and preserve orbital function. In certain instances, adjunctive and/or transorbital approaches can aid in optimizing hemostasis and safeguarding orbital structures.

The purpose of this article is to describe a multidisciplinary adjunctive orbital technique for anterior ethmoidal artery (AEA) ligation and intraoperative protection of orbital contents that is performed in conjunction with oculoplastic surgery, with the goal of enhancing the rhinologist's ability to achieve complete tumor resection while minimizing blood loss and preserving orbital structures through 2 illustrative cases.

## Materials and Methods

### Patient 1

**Figure 1.**
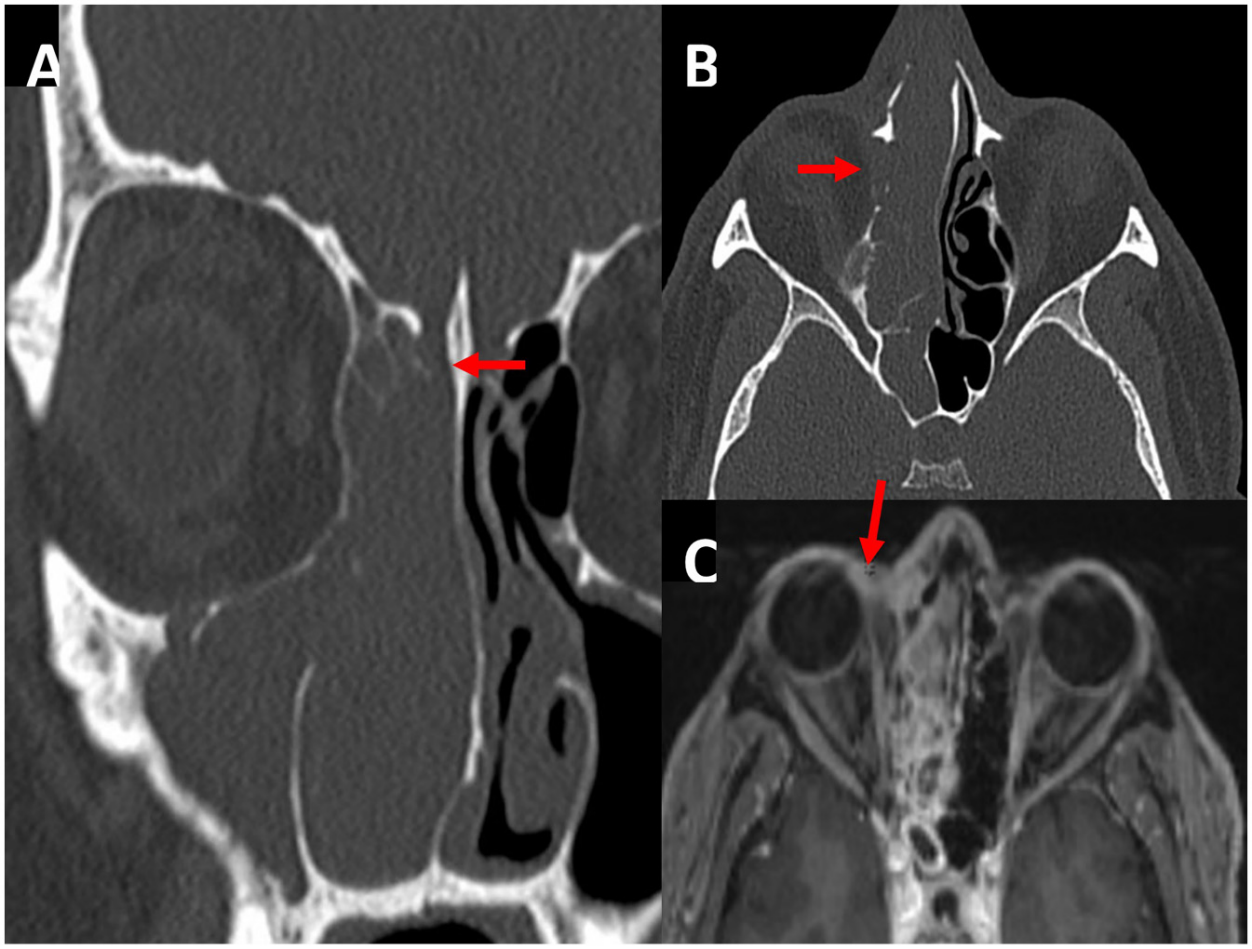
Computed tomography demonstrating potential thinning at the right cribriform plate in (A, red arrow) versus volume averaging and demineralization of the lamina papyracea with concern for extension of disease into the periorbital tissue (B, red arrow). Axial T1 postcontrasted magnetic resonance image slice depicting soft tissue involvement abutting the lacrimal sac (C, red arrow).

A 53-year-old male presented with biopsy-proven squamous cell carcinoma of the ethmoid sinuses. Imaging showed a right nasal cavity mass with thinning of the right cribriform plate (Figure 1A) and lamina papyracea (Figure 1B) with concern for lacrimal sac involvement (Figure 1C).

### Patient 2

**Figure 2.**
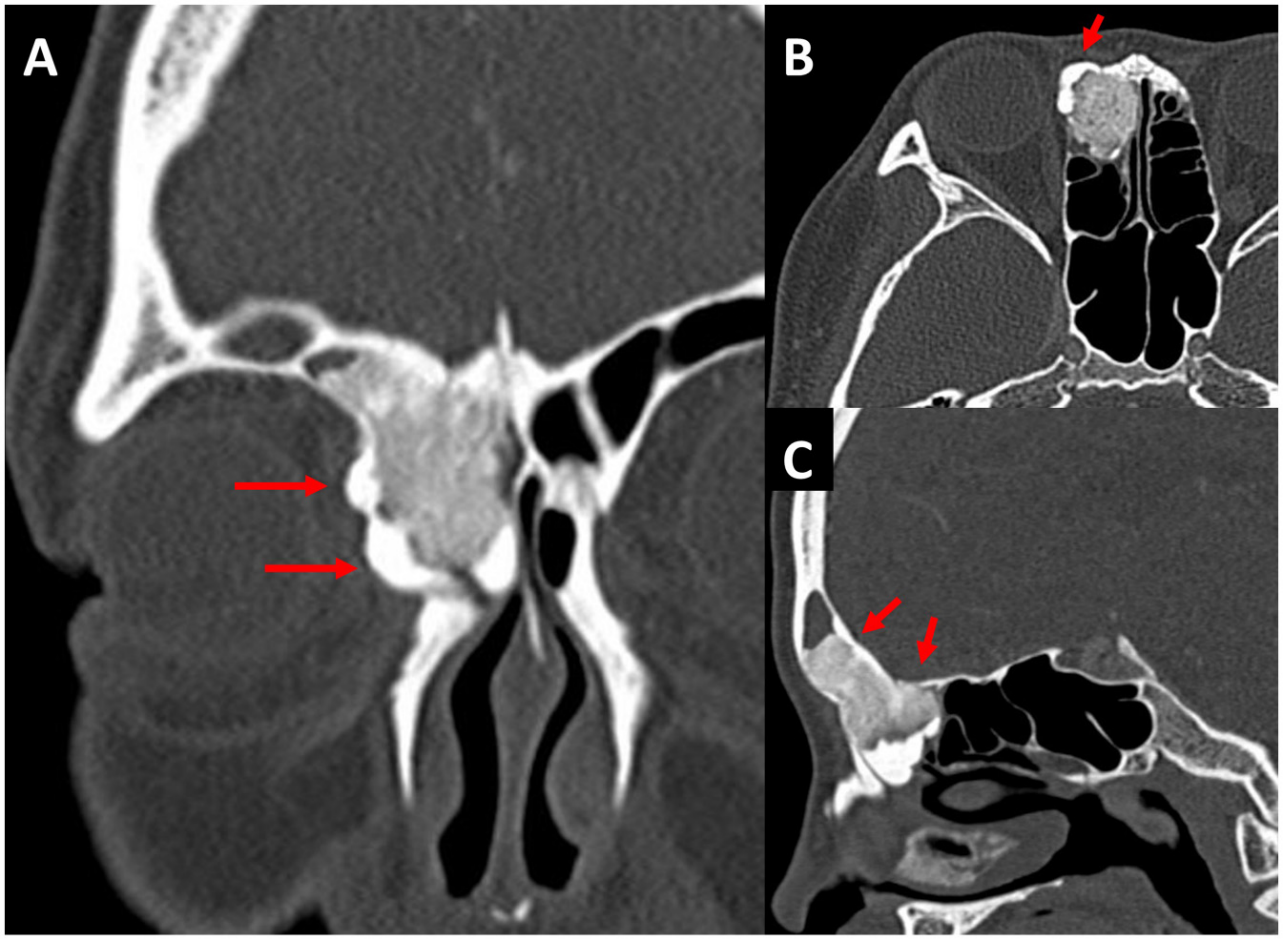
Coronal (A) computed tomography (CT) demonstrates large right sinonasal osteoma with apparent deformity of the right medial orbital wall (arrows) with mass effect on orbital structures. Axial slice (B) noting area of palpable bony protuberance to medial orbit palpated on examination (arrow). Sagittal cut through right paranasal sinuses demonstrating near complete filling of frontal sinus and anterior ethmoid involvement (C) by osteoma with broadly based skull base attachment (arrows).

A 21-year-old female presented with progressive right-sided headaches with nasal endoscopy notable for a submucosal mass in the frontal recess. Imaging demonstrated a large mixed fibrous and osseous lesion within the right frontal recess with extension into the right frontal and anterior ethmoid cells with lateral bulging into the medial orbit consistent with an osteoma (Figure 2).

### Surgical Technique

To perform the transcaruncular approach, an incision is made through the lateral 1/3 of the caruncle and extended superiorly/inferiorly to the level of the upper/lower puncta. The retrocaruncular fascia is entered and dissection is carried medially, accessing the medial orbital wall just posterior to Horner's muscle at the level of the posterior lacrimal crest. During dissection, it is important to remain within this plane to minimize the risk of injury to nearby structures, particularly the lacrimal system. Once the posterior lacrimal crest is visualized, dissection is performed in either a supra- or subperiosteal plane. The AEA is encountered ∼24 mm posterior to the anterior lacrimal crest.

**Figure 3.**
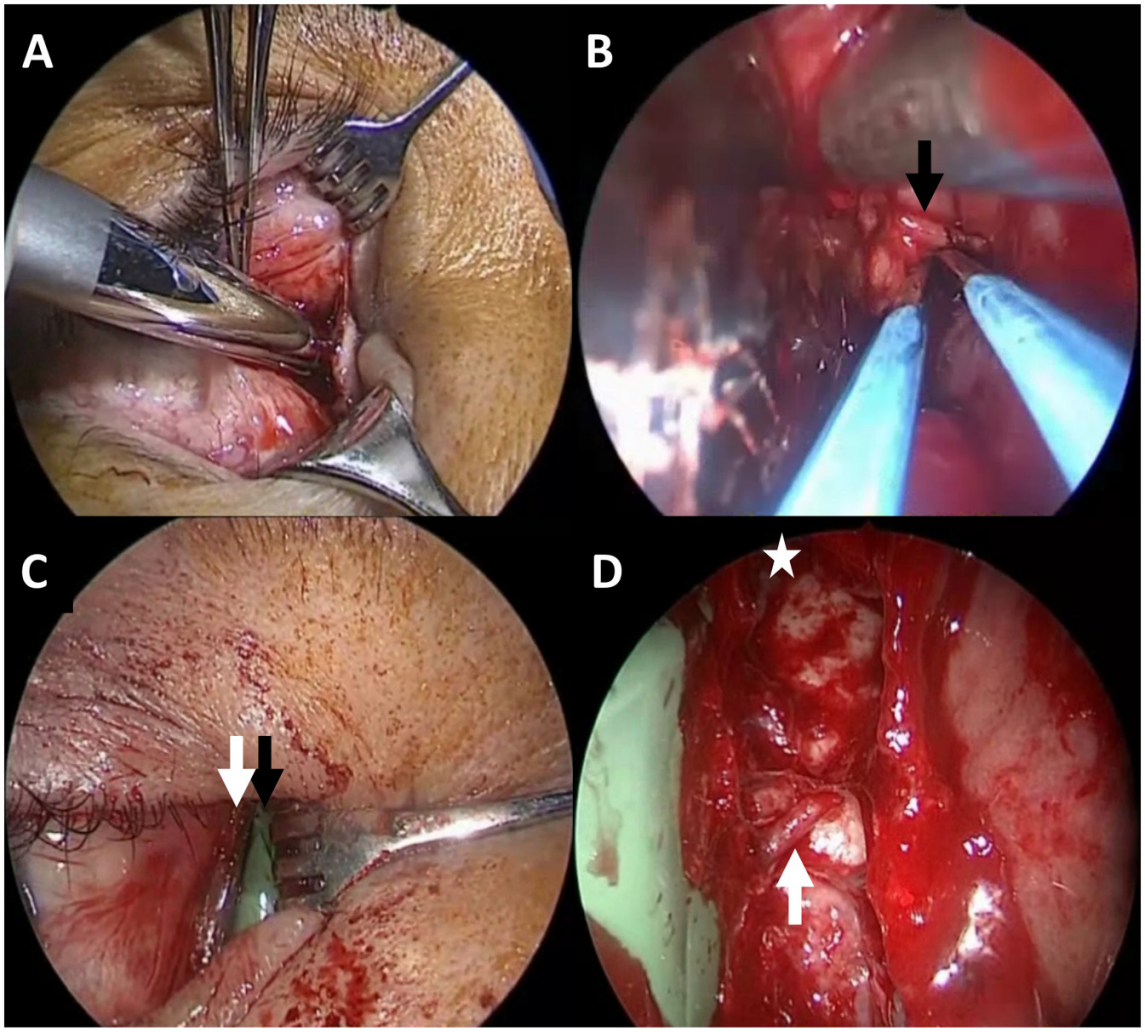
(A) The initial transcaruncular incision beginning at the lateral 1/3 of the caruncle and carried posteriorly. The anterior ethmoid artery is then encountered (B, black arrow) that is controlled by bipolar electrocautery and sharply divided. Once dissection is completed and tumor separated from the orbital contents, we recommend simultaneous placement of the nylon sheet (C, white arrow) that will be used for reconstruction and green shield (C, black arrow) placement to provide a visual barrier for subsequent endonasal dissection. (D) An endonasal view with a 70-degree endoscope. The remnant of the anterior ethmoidal artery is denoted by the white arrow with the frontal sinus at the white star. Note the visible green shield separating the orbital contents for safer dissection.

In patient case 1, a transcaruncular incision was made to allow access to the medial wall of the orbit (Figure 3A). The AEA was identified, cauterized, and divided (Figure 3B). Tumor was then dissected away from the medial wall of the orbit and lacrimal sac. A dacryocystectomy (DCR) was performed and negative frozen margins were obtained. Should the nasolacrimal sac been positive for tumor, a medial maxillectomy with removal of the intraosseous nasolacrimal duct would have been performed. A nylon sheet and green shield were placed between the orbit and tumor tissue/medial orbital wall to aid in protecting the orbit and visualizing orbital boundaries during the sinonasal resection (Figure 3C).

During endonasal resection, the protective shield was readily visible, preventing prolapse of orbital fat into the surgical field and safeguarding the orbital contents (Figure 3D). Following tumor resection, a transconjunctival DCR was performed through the same incision. The nylon sheet was repositioned within the bony orbital defect, with stable bone on all edges and seated within the subperiosteal space to reconstruct the orbital floor and medial wall. Forced ductions were performed followed by medial canthal and conjunctival reconstruction. The medial canthus was secured to stable tissue in the posterior superomedial direction to prevent telecanthus with 5-0 polyglactin suture and the caruncle closed with 6-0 fast gut.

For patient 2, an identical initial approach was performed. To further aid in resection, the periorbita with overlying shield was retracted laterally to facilitate osteoma thinning and removal. A modified Lothrop was then performed, and the remainder of the tumor was removed.

## Results

Video 1 demonstrates the transcaruncular approach to AEA ligation with orbital protection. Both patients recovered well from their surgery. Patient 1 had transient epiphora 7 months after completion of his radiation therapy, requiring a revision DCR. Patient 2 experienced transient diplopia due to perioperative inflammation of the superior oblique that resolved within 1 month.

## Discussion

The transcaruncular approach can efficiently access the medial orbit, allowing for AEA ligation and insertion of a shield for intraoperative protection of orbital contents during sinonasal resection. This approach allows for direct inspection and/or biopsy of the periorbita to assess for tumor invasion. Finally, this pre-emptive dissection enhances the ease of medial wall reconstruction and allows for adjunct procedures such as DCR.

Cadaveric studies have previously described transcaruncular AEA ligation as an adjunct to endoscopic management of sino-orbital disease.^1^ Recently, this technique has been suggested for use in cases of recalcitrant epistaxis.^2^ From our experience, AEA ligation significantly improves intraoperative hemostasis and visualization, allowing for more efficient tumor resection.

The transcaruncular approach has been described as a technique for accessing the medial orbital wall and apex.^3^ In cases requiring additional posterior dissection, the posterior ethmoidal artery can also be ligated. A colored plastic shield placed between the orbital soft tissue and the lamina papyracea provides protection of the orbital contents and visual feedback to aid in soft tissue plane localization during endonasal resection. This would be contraindicated in cases of active cicatrizing conjunctival disease at the time of surgery.

## Conclusion

Minimally invasive orbital approaches to AEA ligation improve hemostasis during sinonasal tumor resection. Shield insertion along the medial orbital wall provides intraoperative protection of the orbital contents, allowing for safer removal of lesions irrespective of lamina papyracea integrity.

## Supplemental Material

**Figure:** Video 1.
